# High-Quality Draft Genome Sequence of *Pseudomonas syringae* pv. Syringae Strain SM, Isolated from Wheat

**DOI:** 10.1128/genomeA.00610-13

**Published:** 2013-08-15

**Authors:** Alexey Dudnik, Robert Dudler

**Affiliations:** Institute of Plant Biology, University of Zurich, Zurich, Switzerland

## Abstract

*Pseudomonas syringae* is one of the most widespread plant pathogens that can cause significant damage to crop plantations. Here, we announce a noncontiguous finished genome sequence of *Pseudomonas syringae* pv. syringae strain SM, isolated from hexaploid wheat. The genome sequence revealed the smallest described complement of type III effectors.

## GENOME ANNOUNCEMENT

*Pseudomonas syringae* strains have been isolated from >180 host species across the entire plant kingdom, including many agriculturally important crops (1). The observed wide host range is reflected in a relatively large genetic heterogeneity among different pathovars. This is most pronounced in the complement of virulence factors, which is also assumed to be the key factor defining the host specificity (2). *Pseudomonas syringae* pv. syringae strain SM was isolated from hexaploid wheat (*Triticum aestivum*) in the United States (3). The strain, which was also denoted D20, has been used in several studies addressing the issue of bacterium-induced systemic resistance in plants (3–7) but never as an infection model for wheat.

A 3-kb paired-end library was generated and sequenced at the Functional Genomics Center Zurich on a Roche genome sequencer FLX+ platform. A total of 974,051 quality filtered reads with a total of 213,333,037 bases were obtained, resulting in 34.8-fold average sequencing coverage. The obtained reads were further *de novo* assembled using Newbler 2.5.3 into 64 contigs combined into one 6.08-Mb-long superscaffold and 3 smaller scaffolds (46.5 kb, 9.09 kb, and 5.24 kb in size). The largest of the minor scaffolds turned out to be a pPT23A family plasmid, the 9-kb scaffold showed sequence similarity to nonribosomal peptide synthase (NRPS) modules, and the smallest scaffold constituted an rRNA operon. A portion of intrascaffold gaps was closed by sequencing of PCR products using Sanger technology, decreasing the total number of contigs to 26. However, it was not possible to precisely map the 9-kb scaffold, but due to its insignificance to the project, it was excluded from the assembly. Initial open reading frame (ORF) prediction and functional annotation were performed using the RAST server (8). The start codons of all the predicted ORFs were further manually verified using the position of potential ribosomal binding sites and BLASTp (9) alignments with homologous ORFs from other *Pseudomonas* strains as a reference. Functional annotations were also refined for every ORF using BLASTp searches against the nonredundant protein sequence database (nr) and the NCBI Conserved Domain search engine (10).

The estimated genome size of strain SM is 6,124,102 bp, with an average G+C content of 58.73%. It contains 5,072 protein-coding sequences (excluding pseudogenes), five rRNA operons, and 64 tRNA genes for all of the amino acids. Notably, it contains a complete type III secretion system and seven known effector proteins: AvrE1, HopAA1, HopI1, HopM1, HopBA1, HopA2, and HopAZ1. In addition, there are two complete type VI secretion system gene clusters and 12 putative effector proteins belonging to the VgrG and Hcp1 families, as well as intact gene clusters for the biosynthesis of syringopeptin and mangotoxin. All of these genome components have previously been demonstrated to be involved in virulence and epiphytic fitness of *P. syringae*, as well as in competition of pseudomonads with other microbial species (11–16).

### Nucleotide sequence accession numbers.

This whole-genome shotgun project has been deposited at DDBJ/EMBL/GenBank under the accession no. APWT00000000. The version described in this paper is the first version, APWT01000000.
